# Trafficking in blood vessel development

**DOI:** 10.1007/s10456-022-09838-5

**Published:** 2022-04-21

**Authors:** Caitlin R. Francis, Erich J. Kushner

**Affiliations:** Department of Biological Sciences, University of Denver, Denver, CO 80210 USA

**Keywords:** Angiogenesis, Blood vessel, Development, Vascular, Endothelial, Endothelium, Lumen formation, Sprouting, Trafficking, Exocytosis, Endocytosis, Recycling, Junctions

## Abstract

Blood vessels demonstrate a multitude of complex signaling programs that work in concert to produce functional vasculature networks during development. A known, but less widely studied, area of endothelial cell regulation is vesicular trafficking, also termed sorting. After moving through the Golgi apparatus, proteins are shuttled to organelles, plugged into membranes, recycled, or degraded depending on the internal and extrinsic cues. A snapshot of these protein-sorting systems can be viewed as a trafficking signature that is not only unique to endothelial tissue, but critically important for blood vessel form and function. In this review, we will cover how vesicular trafficking impacts various aspects of angiogenesis, such as sprouting, lumen formation, vessel stabilization, and secretion, emphasizing the role of Rab GTPase family members and their various effectors.

## Introduction

Blood vessels are the earliest organ system to arise in development owing to their absolute requirement for transport of oxygen and nutrients to growing tissues. Angiogenesis is the proliferation of previously established blood vessels through a variety of highly regulated programs. Understanding how angiogenesis works has had, and continues to have, tremendous medical value. Although, our understanding of these intricate cell autonomous and tissue-wide programs is still in its infancy on many levels. Recent advances in RNA-Seq and single-cell RNA-Seq have allowed investigators to map complex transcriptional networks with unprecedented resolution; however, we are finding these networks do not entirely reprise the full phenotypic picture.

As taught in entry level biology, mRNA translation is only the start of a protein's journey to becoming functional. Proteins are modified through post-translational modifications and are typically processed through the Golgi apparatus, packaged into vesicles, and then delivered to a precise intracellular location(s). Perturbations or regulatory signaling at any of these steps can have profound consequences on tissue morphogenesis. Moreover, many of these post-Golgi steps can be completely divorced from traditional transcriptional feedback loops; thus, trafficking programs can be self-regulating with little transcriptional input. The purpose of this review is to highlight and expound on emerging roles of trafficking in endothelial biology. We contend that both endothelial-specific as well as more ubiquitous trafficking signatures need to be mapped to truly understand angiogenesis during development and in disease. In the following sections, we have broken various components of angiogenesis down by function and discuss the relevant trafficking programs. In many instances there are no endothelial studies to draw upon, in this case we infer function from experiments performed in other systems.

## Trafficking and Rab GTPase proteins

Trafficking is a general term referring to vesicular transport of proteins that encompasses an intricate delivery network between organelles and distinct intracellular compartments. This vesicular transport system moves newly made proteins from the endoplasmic reticulum (ER) to the Golgi apparatus, and then from the Golgi to its terminal destination. In this review, we will primarily focus on post-Golgi and endosomal transport, as the mechanisms that transfer proteins from the ER to the Golgi complex are fairly-well conserved, while post-Golgi and endocytic trafficking events demonstrate greater organotypic heterogeneity. At the distal trans-Golgi network (TGN), both membrane-bound and cytosolic proteins are packaged into vesicles of different shapes and sizes depending on the vesicular cargo and destination. Here, a variety of factors will decorate the nascent vesicle to aid its physical transport, such as kinesin and dynein [1], as well as to designate its trafficking route, or pattern (as vesicle carriers may have many routes). In this vein, trafficking can be described as exosomal (inside to outside), endosomal (outside to inside) and recycling (switching between exosomal and endosomal). For example, vascular endothelial growth factor receptor-2 (VEGFR2) likely emerges from the Golgi complex to the plasma membrane via an anterograde route. Thereafter, the receptor is plugged into a recycling holding pattern, being cyclically internalized and inserted back into the membrane [2]. Upon ligand binding, VEGFR2 is endocytosed and degraded via the lysosome [3]. In this example, it is obvious that alterations in any trafficking step of an important protein such as VEGFR2 would have profound effects on blood vessel function independent of transcriptional activity. As many proteins are distinct to endothelial tissues, it would also be predicted that blood vessels would exhibit unique trafficking signatures. The question then becomes what factors contribute to unique endothelial-specific trafficking patterns during blood vessel development(?); and how are they themselves regulated?

The answer to these questions is almost entirely contingent upon the biological function being performed. However, a family of proteins that dictate trafficking hold great promise for deciphering endothelial trafficking signatures. In this regard, Rab GTPases are central to eukaryotic cell membrane-trafficking events as they function in vesicle formation, motility, membrane-tethering, as well as docking and fusion events [4]. Rab proteins are capable of recruiting motor proteins, triggering scaffold formation and coding compartment identity [5, 6]. A common analogy is that Rab proteins work as an intracellular barcoding system. Each unique Rab acts as a barcode for vesicular cargo, orchestrating the carrier route. Looking across tissue types, a single Rab can have several types of vesicular cargos (e.g. distinct intracellular vesicles). Conversely, a particular vesicle can be handed off between several Rab family members during its life-cycle (e.g. multiple barcodes). This interplay between activated Rabs and their downstream interacting proteins, termed effectors [7], can be dynamically altered by both intrinsic and extrinsic stimuli for rapid cellular responses.

There are more than 70 Rab GTPases identified in humans, which makes the Rab family the largest of the monomeric small GTPases [8]. The regulatory principle of Rab GTPases is based on the interconversion between an active (GTP-bound) and an inactive (GDP-bound) state. This conformational oscillation is managed by other proteins such as activating guanine exchange factors (GEFs) and deactivating GTPase-activating proteins (GAPs) [8]. In an active state, Rab GTPases interact with unique effectors to carry out their various roles. To date, many Rab-effector proteins have been identified in non-endothelial cell types; however, many remain to be characterized with regard to blood vessel function (Table 1). There are many excellent reviews that go into greater detail about Rab GTPase evolution, function and regulation some of which are cited here [8–11]. The heterogeneity of Rab function across different cell types highlights the importance of investigating Rabs unique tissue behaviors. In blood vessels, how Rab GTPases and their associated effector(s) contribute to blood vessel formation and homeostasis remains incompletely understood. This is unfortunate, not only for garnering a more comprehensive understanding of trafficking in endothelial biology, but for the potential missed opportunities to develop novel disease therapeutics.

**Table 1:** Rab GTPase’s regulators, effectors and function

Rab GTPase	GEF(s)	GAP(s)	Effector	Function	Citations
Rab1a/b	TRAPP I, DrrA	TBC1D20	–	ER to Golgi trafficking	Lamber et al., *Current Opinions in Cell Biology*, 2019
Rab2b	–	–	Bicaudal-D, RUND-1, CCCP-1	ER to Golgi trafficking	Zhen et al., *Journal of Cell Science*, 2015; Ailion et al., *Neuron*, 2014
Rab3d	–	–	–	WPB localization	Zografou et al., *Journal of Cell Science*, 2012
Rab4a/b	–	TBC1D11, EVI5-like	Rabip4, Rabaptin-5, RabEP2	Early-endosome trafficking	Stein et al., *Advanced Drug Delivery Reviews*, 2003; Zografou et al., *Journal of Cell Science*, 2012
Rab5a/b/c	Rabex-5 (Vps9),, Rabaptin-5	TBC1D3/RUTBC3/USP6NL	EEA1, RIN2	Early-endosome trafficking, podxl trafficking in epithelium	Stein et al., *Advanced Drug Delivery Reviews*, 2003; Richards et al., *Current Biology*, 2015
Rab6	RIC1-RGP1	–	Bicaudal-D	Golgi-localized trafficking	Lamber et al., *Current Opinions in Cell Biology*, 2019; Zhen et al., Journal of Cell Science, 2015
Rab7	MON1-CCZ1	TBC1D5	RILP, VPS34, HOPS	Lysosome transport	Lamber et al., *Current Opinions in Cell Biology*, 2019; Stein et al., *Advanced Drug Delivery Reviews*, 2003; Zhen et al., *Journal of Cell Science*, 2015
Rab8a	Rabin-8/GRAB/Mss450/C9Orf72	TBC1D1/TBC1D30/TBC1D4	–	TGN trafficking	Müller et al., *Small GTPases*, 2018
Rab9	–	–	RUTBC1, RUTBC2	Late endosome	Zhen et al., *Journal of Cell Science*, 2015
Rab10	DENND4c	TBC1D1/TBC1D4/EVI5-L		Basolateral trafficking	Gross et al., *Angiogenesis*, 2021
Rab11a/b	SH3BP5 (REI-1)/SH3BP5 (REI-1)	TBC1D11/TBC1D15/EVI5, TBC1D14	Rip11, RCP, Eferin, Protrudin	Endocytic uptake and recycling	Stein et al., *Advanced Drug Delivery Reviews*, 2003; Zhen et al., *Journal of Cell Science*, 2015
Rab13	DENND1c	TBC1D10A, TBC1D25	–	Tubular endosome, TGN trafficking	Müller et al., *Small GTPases*, 2018; Homm, et al., *The FEBS Journal*, 2021
Rab14	DENND6	TBC1D1	–	Early endosome trafficking	Müller et al., *Small GTPases*, 2018; Homma et al., *The FEBS Journal*, 2021
Rab15	–	–	–	WPB localization, endosomes in other tissues	Zografou et al., *Journal of Cell Science*, 2012
Rab21	–	–	Protein tyrosine phosphatase receptor type F (PTPRF)	Endocytosis of integrins bound to fibronectin	Mana et al., *Nature Communications*, 2016
Rab25	–	–	–	Podxl trafficking in epithelium	Richards et al., *Current Biology*, 2015
Rab 27	MADD/DENN/Rab3GEP	TBC1D10A/EPI64/Rab27‐GAPα, TBC1D10B/FLJ13130	Slp2a, MYRIP, Slp4a	WPB negative regulator	Francis et al., *ATVB*, 2021; Fukuda et al., *Traffic*, 2013
Rab33	RIC1-RGP1	RUTBC1, RUTBC2	–	–	Lamber et al., *Current Opinions in Cell Biology*, 2019; Zografou et al., *Journal of Cell Science*, 2012
Rab35	DENND1a/DENND1b/DENND1c	TBC1D10A/TBC1D10B/TBC1D10C/TBC1D13/TBC1D24	ACAP2, RUSC2, OCRL, MICAL-L1	Plasma membrane endocytosis, cytoskeletal re-arrangements	Chaineau et al., *Traffic*, 2013; Marat et al., *Molecular Biology of the Cell*, 2012
Rab37	–	–	–	WPB localization	Zografou et al., *Journal of Cell Science*, 2012
Rab46	–	–	–	WPB localization	Miteva et al., *Journal of Cell Biology*, 2019

*Podxl* Podocalyxin, *WPB* Weibel–Palade body, *TGN* trans-Golgi network

## Sprouting angiogenesis

A primary cellular function during angiogenesis entails endothelial cell(s) sprouting from a parent vessel, typically in response to extrinsic growth factors. For these events, we are referring to a tip cell that would be leading several stalk cells in a canonical tip/stalk cell hierarchy [12]. During this process endothelial cells are sensing growth factor ligands that rearrange cell polarity, promote actin dynamics and integrin-based cell motility programs, and break-down extracellular matrix, ECM [13–17]. A primary initiator in this event would be an endothelial cell binding a growth factor, namely vascular endothelial growth factor (VEGF) on its cognate VEGFR2 receptor. Receptor endocytosis, particularly VEGFR2 internalization, is an excellent example of how trafficking can mediate endothelial function. This event is likely the most well-studied trafficking-related program in endothelial biology today. As such, there are several reviews that go into detail cited here [2, 18–20]; thus, we will cover more recent data related to this phenomenon.

**Fig. 1:**
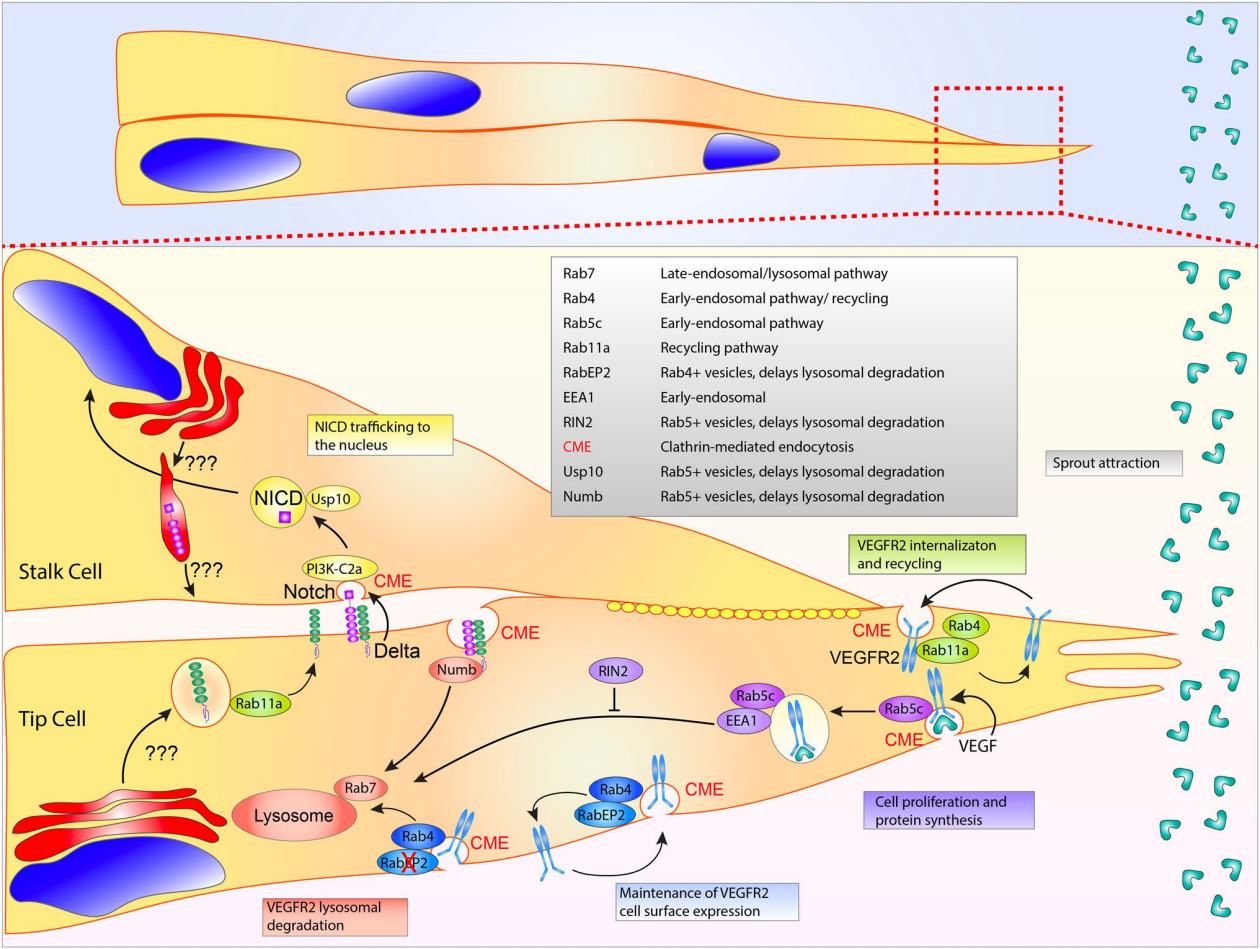
Sprouting angiogenesis and Notch trafficking. Sprout migration is dependent on vascular endothelial growth factor receptor 2 (VEGFR2) endocytosis. Upon vascular endothelial growth factor (VEGF) ligand binding, Rab5c and early-endosome antigen 1 (EEA1) decorate the internalizing clathrin-coated pit. RIN2 prevents lysosomal degradation of the Rab5 positive vesicles. VEGFR2 cell surface expression is maintained by both Rab11a and Rab4 recycling. Rab4 aids in maintaining VEGFR2 expression. In the absence of RabEP2, VEGFR2 is transitioned to a Rab7-positive vesicle destined for lysosomal degradation. During Notch and delta-like ligand 4 (Dll4) binding, Dll4 pulls on the Notch receptor using clathrin-mediated endocytosis (CME) allowing for S2 and S3 cleavage events. Thereafter, the released Notch extracellular domain is transcytosed into the Dll4 presenting cell and presumably degraded. The Notch intracellular domain (NICD) is subsequently protected from proteosomal degradation in transit to the nucleus by the deubiquitinase Usp10. Anterograde trafficking of Notch and Dll4 to the plasma membrane is incompletely understood. Table lists proteins depicted in figure with corresponding function

Internalization of VEGFR2 is initiated through clathrin-mediated endocytosis, CME [21, 22] in which the receptor is removed from the plasma membrane and internalized in the form of a vesicle. In the inactive, non-ligand bound state, VEGFR2 is plugged into a Rab4a or Rab11a-mediated recycling pathway, continuously being internalized and returned to the plasma membrane [23, 24] (Fig. 1). There is some data supporting a clathrin-independent pathway, such as caveolin-dependent endocytosis, in receptor internalization [25]; however, recent literature has significantly shifted away from the notion that caveolae participate in endocytic processes, but are primarily membrane reservoirs, buffering changes in membrane tension during cellular dynamics [26, 27]. Upon ligand binding, newly endocytosed VEGFR2-positive vesicles are marked with Rab5 and early endosome antigen-1 (EEA1). Rab5, is most associated with endocytic events and receptor tyrosine kinase internalization [28, 29]. Rab5-positive early endosomes are transitioned to a Rab7 late endosome and targeted to the lysosome for destruction [30, 31]. Receptor internalization and degradation will reduce the amount of naïve cell-surface receptors, this in turn, will limit the signaling potential of the ligand. This pathway is in no way unique to VEGFR2 as many other receptor tyrosine kinases [32] demonstrate a similar mode of endocytic regulation [33, 34]. There is some controversy if receptor endocytosis, per se, is required for downstream VEGFR2-related signaling. Several investigations have shown that loss of CME blunts downstream VEGFR2 signaling [35, 36], while others report that loss of CME does not dampen signaling potential [22, 37]. In terms of sprouting, any program that alters growth factor signaling duration and amplitude will elicit a profound effect on downstream cellular behaviors.

Recently, several groups have identified additional trafficking determinants involved in VEGFR2 endocytosis. VEGFR2’s insertion into a recycling pathway on face-value would seem to be more energetically costly than a unidirectional trafficking event where the receptor is statically plugged into the membrane, primed for ligand binding. However, constitutive recycling of VEGFR2 plays a protective role against receptor shedding. Inhibition of CME will increase shedding of the VEGFR2 ectodomain, indicating that endosomal recycling is important for receptor plasma membrane retention [38]. Using a screen against Rab GAPs, TBC1D10A-C was flagged for its impact on endothelial VEGFR2 signaling, tube formation and cell migration [18]. Here, the authors show the same GAP family members can elicit contrary responses in terms of VEGFR2 signaling, one decreasing downstream ERK activation, while the other enhancing it. This is likely related to each GAPs unique affinity to a particular Rab or group of Rabs. In this case, TBC1D10A has affinity for Rab13, interestingly this has also been shown to be a GAP for Rab27a and Rab35 [39, 40]. In another investigation focused on VEGFR2 endocytosis the authors demonstrated that the protein RabEP2 partners with the recycling Rab4 to maintain VEGFR2 cell surface expression. In the absence of RabEP2, Rab4-positive vesicles were diverted to a Rab7 lysosomal pathway, significantly attenuating VEGF signaling [41]. It was also reported that Rab5c partners with RIN2 to delay lysosomal degradation to increase downstream VEGFR2 signaling [3]. In this article, loss of RIN2 or Rab5c-mediated endosomal stabilization blunted VEGFR2 signaling of Akt and ERK leading to defects in sprouting parameters in culture and zebrafish blood vessel development. These reports nicely illustrate how critical endothelial-specific signaling can be fine-tuned by endosomal processes.

An interesting point here is uncoupling Rab-mediated effects on endothelial cell migration from their interactions on the VEGF or other growth factor signaling. For instance, some have purported that knocking out a particular Rab affects endothelial migration [42–44]; although this is undoubtedly the case, the primary defect is connected to VEGFR2-related viability and chemotaxis, not a direct effect on machinery involved in endothelial cell motility. In this case, there are few studies directly exploring endothelial trafficking factors that influence cell motility, per se. In a candidate screen directed against Rab3a, Rab3b, Rab8a, Rab11a, Rab27a, RalA, RalB and caveolin-1 investigating endothelial tube formation, it was observed that a variety of the Rab GTPases reduced sprouting behaviors [45], suggesting an effect on cell motility programs in some cases; although, the mechanisms for these perturbations were not described. There are many reports that directly test the role of cytoskeletal regulators in endothelial tissues, but few that identify how trafficking regulators interface with these systems. Future research coupling both trafficking and cytoskeletal signaling networks would be important as endothelial cells look and move (collectively and individually) differently from epithelial cells in which the bulk of this type of research has been published.

Integrins are extracellular receptors that engage the ECM and are highly involved with cell migration and general apicobasal polarity [46]. These receptors are part of a large complex called a focal adhesion that links the actin cytoskeleton to the ECM generating the propulsive force to move a cell, or collectively, a sprout [42]. As part of a cyclical process, integrins are continually recycled, placed on the basal cell membrane, anchored to the ECM and endocytosed as the cell propels itself forward [47]. Trafficking factors have been shown to dramatically affect cell migration through regulating the availability of integrin receptors in endothelial cells. For instance Rab21 with protein tyrosine phosphatase receptor type f has been reported to endocytose α_5_β_1_ integrins bound to fibronectin [48]. The cytoskeletal regulator RhoJ has been shown the regulate endocytic processes including α_5_β_1_ integrin trafficking [49]. Similarly, Arf6 has been shown to be a potent activator of integrin recycling across many cell types, controlling both fast and slow integrin treadmilling [50–52]. Arf6 influences CME as well as recycling, interfacing with Rab11a [53]. Loss of Arf6 and downstream perturbations in integrin activation can have a profound effect on sprouting angiogenesis [54].

## Cell–cell junction regulation

Junctional regulation is paramount to physiological blood vessel development. Individual endothelial cell junctions must work in concert to stabilize or loosen cell–cell connections by differentially recruiting or removing junctional proteins. In endothelial cells, a major junction protein of interest is VE-cadherin. VE-cadherin is an endothelial-specific adherens junction and several excellent reviews on its regulation, interactions with the actin cytoskeleton and crosstalk with growth factor signaling are cited here [55–57]. In terms of trafficking two questions are essential: (1) how does VE-cadherin arrive at basolateral junctions (?); and (2) how is it destabilized during sprouting morphogenesis? Once at the plasma membrane VE-cadherin is likely plugged into a Rab11a recycling pathway as knockout of the Rab11a has been shown to decrease endothelial barrier function [58]. Similarly, it has been reported that Rab11a directly binds VE-cadherin [59]. This data would suggest that VE-cadherin is plugged into a recycling loop similar to RTK receptors. This finding is congruent with Rab11a-based E-cadherin trafficking in epithelial cells [60]. However, caution should be taken when ascribing direct function to Rab11a recycling as so many peripheral trafficking programs leverage this network.

**Fig. 2:**
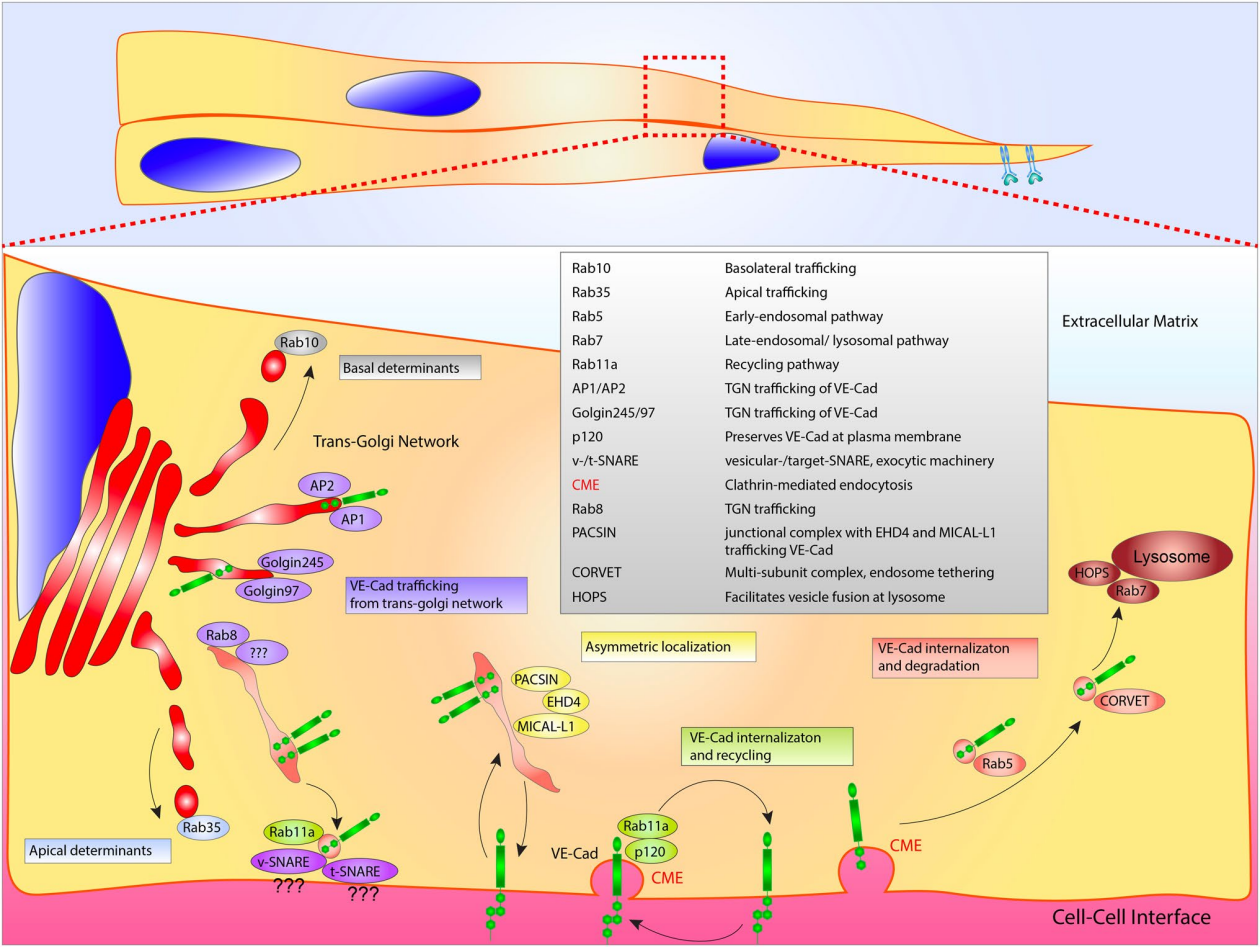
VE-cadherin trafficking regulation. VE-Cadherin (VE-Cad) trafficking from the Golgi apparatus to the plasma membrane is potentially aided by AP1, AP2, golgin97 and golgin245. Post-Golgi transporter Rab8 is positioned at the trans-Golgi network, where it may be involved in trafficking to the plasma membrane. At the plasma membrane exocytic machinery, such as vesicular (v)-SNARE’s and tethering (t)-SNARE’s play a role in vesicle capture and docking. Once plugged into the plasma membrane, VE-Cad is maintained in a recycling loop via Rab11a and p120. Asymmetric localization of VE-Cad is thought to involve a PACSIN/EHD4/MICAL-L1 complex. VE-Cad endocytosis may be regulated by Rab5-mediated shuttling to the CORVET and HOPS complex prior to lysosomal degradation. Rab35 and Rab10 act as either apical or basolateral determinants, respectively. Table lists proteins depicted in figure with corresponding function

With regard to the initial anterograde trafficking, how newly translated VE-cadherin is first transported from the Golgi apparatus to junctional complexes is largely uncharacterized. Rab11a is typically a terminal trafficking destination, such that, the early post-Golgi Rab-based mediators that are responsible for delivery of VE-cadherin to Rab11a have not been charted to our knowledge. To this point, Malinova et al. more recently reported a complex involving PACSIN2, EHD4, and MICAL-L1 which influenced VE-cadherin asymmetric localization during sprouting [61] (Fig. 2). In this investigation, PACSIN2 recruited the trafficking regulators EHD4 and MICAL-L1 to the rear of asymmetric adherens junctions. Given this complex has been associated with tubular transport in other tissue types, it could be posited that VE-cadherin is shuttled by Rab6a, Rab8, or Rab10 which have all been shown to interface with MICAL-L1 on tubulated vesicles [62].

Based-off other tissues that have more extensively characterized the related E-cadherin protein, we can infer a few basic pathways. At the TGN, the adaptor proteins AP1 and AP2, co-localize with E-cadherin likely playing a role in biosynthetic sorting [63, 64]. Other proteins, such as golgin-245 and golgin-97, also have been shown to localize with E-cadherin [65]; again, none of these pathways have been explored in endothelial junction assembly. At the plasma membrane, Rab11a-mediated junctional recycling would suggest the presence of tethering(t)-SNARES and vesicle(v)-SNAREs. This class of proteins mediate vesicle fusion, controlling secretion as well as the general constituents of the plasma membrane (e.g. receptors, glycoproteins, etc.); reviews on SNARE biology are cited here [66, 67]. In terms of endothelial function this could be a fertile area of research as the constituents of t- and v-SNAREs can greatly differ by tissue type [68]. Given the 60+ SNAREs work synergistically with the 70+ Rab GTPases to govern trafficking, one could hypothesize endothelial-specific Rab/SNARE combinations would be plausible.

Akin to receptor endocytosis, there is a much richer knowledge base of how VE-cadherin is endocytosed, reducing junctional integrity. VE-cadherin’s cytoplasmic tail contains a juxtamembrane domain (JMD) that interacts with the armadillo protein p120 [69, 70]. P120 is believed to obscure the endocytic signal (DEE) [69], increasing VE-cadherin’s life-time at the membrane. Grimsley-Myers et al. [71] demonstrated that genetic ablation of this motif in mice drastically reduced blood vessel integrity and barrier function. The presumed function is that removal of p120 exposes the conserved DEE domain on the cytoplasmic tail of VE-cadherin. Once exposed, this signals for initiation of CME and entry into the Rab11a-based recycling compartment [72, 73]. Endocytosed VE-cadherin can be either returned to the plasma membrane or targeted for degradation. In the later scenario, the c-terminal catenin-binding domain is cleaved off by calpain, shunting VE-cadherin into a degradative pathway [72]. Rab5a has shown to be critical for this response. Loss of Rab5a significantly reduced VE-cadherin internalization with LPS stimulation [73]. More evidence for this Rab11a to Rab5a degradative trafficking cascade is demonstrated by Rab11a depletion results in blunted VE-cadherin endocytosis, putting Rab11a upstream in this pathway [59].

After entry into the Rab5 early endosome, there is scant information on how VE-cadherin is trafficked. The assumption would be that the Rab5a endosome would engage a multi-subunit tether complex called CORVET (class C core vacuole/endosome tethering) that would reorganize the VE-cadherin-containing vesicle, priming the early endosome for conversion to a Rab7-decorated late endosome [74]. Rab7 classically binds with the homotypic fusion and vacuole protein sorting (HOPS) complex that facilitates fusion to the lysosome [75]. In non-endothelial cell types, depletion of VPS proteins that constitute CORVET or HOPS multi-subunit tethers inhibit lysosome-mediated degradation [76]. Exactly how endothelial cells employ these systems with regard to VE-cadherin regulation, or other endocytosed proteins, has not been studied in any great detail. Given the supple balance of cell–cell adhesion required for proper blood vessel formation, it is tempting to speculate that programs identified in VEGFR2 endocytosis such as RabEP2 and RIN2 could also be operative in this pathway providing extra gradations on the endocytic dial, of sorts.

## Lumen formation

An endothelial cell’s ability to polarize and create a hollow cavity is one of the most notable anatomic characteristics of blood vessels as a tubular fluid transport system. The intrinsic signaling programs that allow endothelial cells to create de novo luminal surfaces are vital to both blood vessel morphogenesis and general function. Trafficking programs play a substantial role in the formation of a new apical membrane (also termed luminal membrane) that will be the plasma membrane surface adjacent to the lumen cavity and later will be in contact with circulating blood constituents. For this review, we will focus on trafficking factors that influence the establishment of the apical membrane during lumen biogenesis. Cytoskeletal factors, principally actin regulating proteins, also play a fundamental role in this process and the following reviews cover this topic in detail [77–80].

**Fig. 3:**
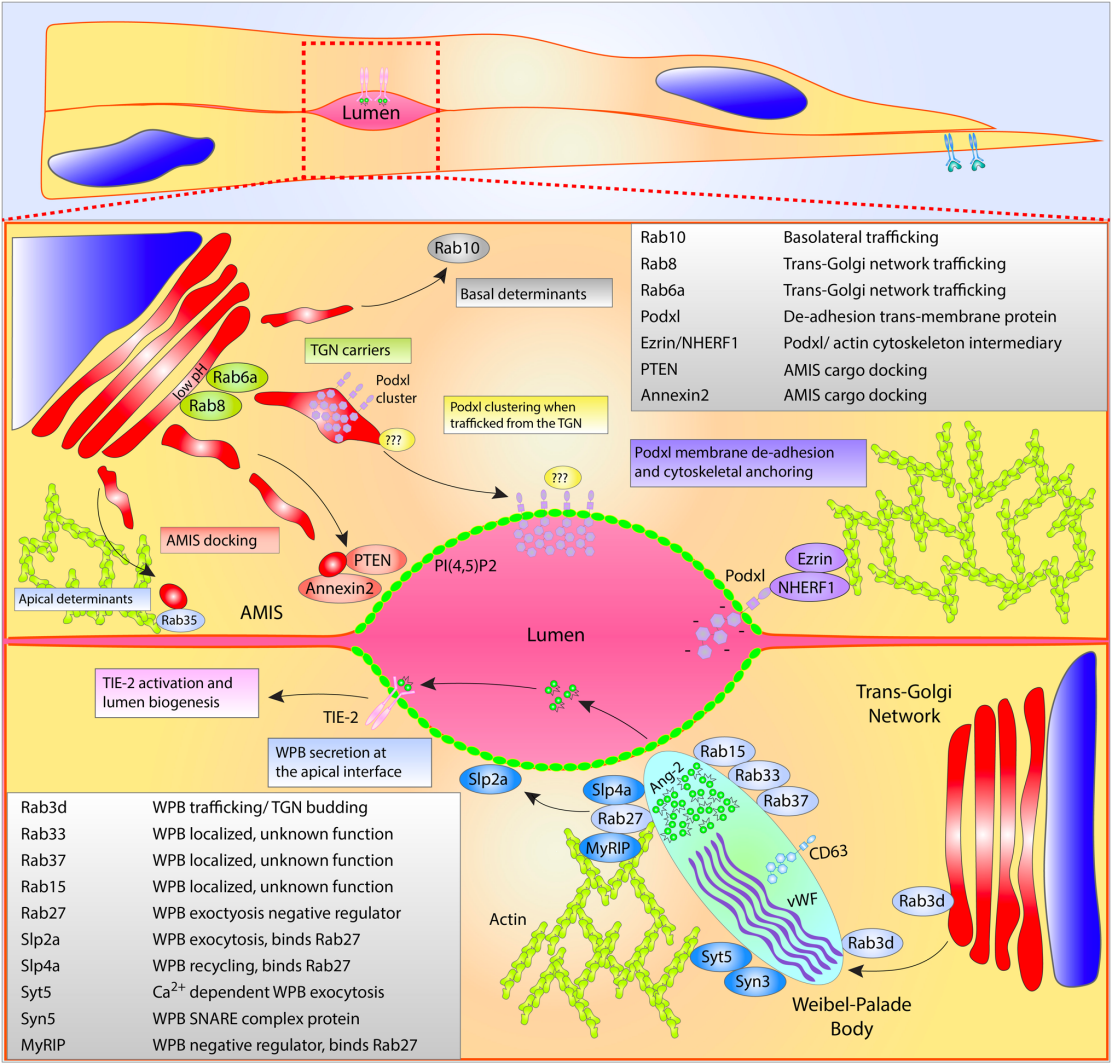
Endothelial lumen formation and secretion. Top cell depicts trafficking of proteins related to lumen formation. From the Golgi complex, apically destined cargo may be transported within Rab6 and Rab8 vesicles or tubular networks. Podocalyxin (Podxl), a required luminal transmembrane protein, may be first recognized at the acidic trans-Golgi network (TGN) via protein clustering aided by addition of carbohydrate moieties. Lipid modification such as PI(4,5)P2 decorate the apical membrane initiation site (AMIS). Once Podxl is deposited into the apical membrane, NHERF1 and Ezrin complex with Podxl and the actin cytoskeleton. Other apical determinants involved in lumenogenesis localize to the AMIS such as Rab35, Annexin2 and PTEN. Bottom cell Weibel–Palade body (WPB) trafficking. Many Rab GTPases have been connected to the trafficking of WPB’s, shown are Rab3d, Rab37, Rab33, Rab15, and Rab27a. Furthermore, exocytic machinery is shown including Syn3, Syt5, Slp4a and Slp2a. MyRIP and Rab27a are negative regulators of WPB secretion sequestering WPBs within the actin cytoskeleton. Secretion of angiopoietin-2 (Ang2) from WPBs causes activation of the TIE-2 receptor and signaling related to lumen formation. Each table lists proteins depicted in figure with corresponding function

**Fig. 4:**
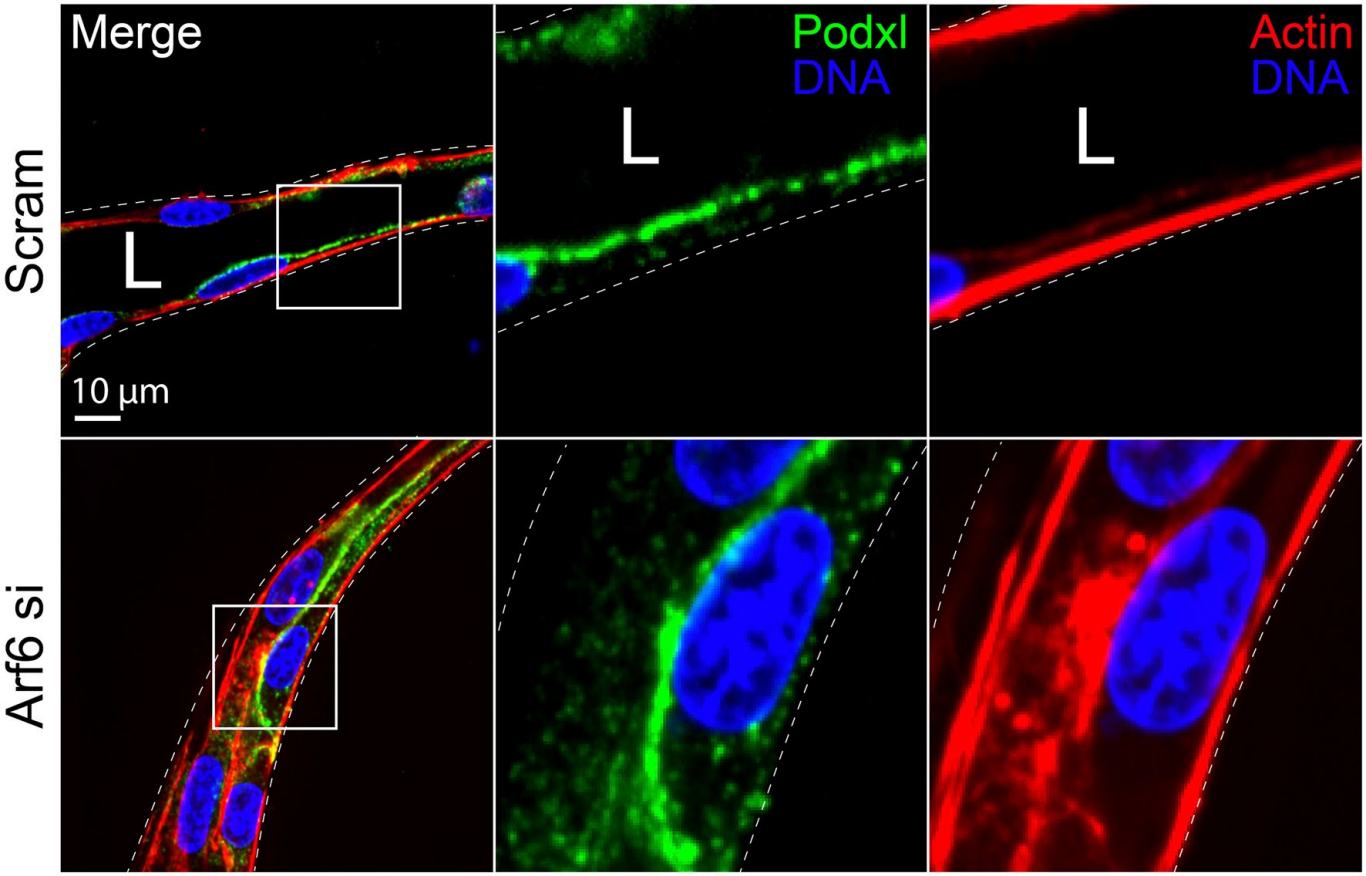
Loss of integrin recycling via Arf6 knockout disrupts lumen biogenesis. Representative endothelial cell sprout treated with scramble (Scram) siRNA (si) or Arf6 si and stained for podocalyxin (Podxl) and actin localization. Note the absence of a luminal cavity upon disruption of integrin signaling via Arf6 knockdown. The white boxes represent areas of magnification. Dotted lines are sprout boundaries. *L* lumen

During lumen initiation a clustering of vesicular deliveries are focused to internal sites of cell–cell contact, this area is termed the apical membrane initiation site, AMIS [81] (Fig. 3). The AMIS location is dependent on both internal cell–cell contacts and basal membrane integrin engagement to provide the cell with a rudimentary polarity cue. This dependency on a polarity axis informed by junctions and ECM engagement is well established as loss of junctional stability and/or integrin signaling in nearly any system significantly precludes lumen formation (Fig. 4) [82]. Once an AMIS is present, it can be presumed that the cell generally has three distinct membrane surfaces, apical, basal and junctional (or basolateral) that exhibit disparate, local signaling and trafficking programs. In endothelial biology, trafficking mediators that participate in AMIS formation are nowhere near as characterized as their epithelial counterparts. This is in large part due to their rectangular shape and spatially segregated apical and basal domains, while ECs are exceedingly flat exhibiting a mesenchymal morphology [83]. In some instances, the distance between the apical and basal domains in ECs are diffraction limited (≤ 500 nm), hindering imaging of either membrane surface. Adding to the complexity, several investigations, including our work, demonstrate that epithelial apical trafficking programs are largely divergent in endothelial cells [84]; thus, this literature should not be viewed as completely interchangeable.

Trafficking directed to the AMIS is first instructed by the presence of various lipid species. For instance, PIP_2_ is one of the earliest apical membrane lipid types being highly enriched at the forming AMIS. PIP_2_ promotes the recruitment of many proteins such as those in the synaptotagmin-like protein family [85]. This protein family also contains a Rab-binding domain to tether Rabs proximal to the apical membrane allowing for fusion of their contents. Our work recently demonstrated that synaptotagmin-like protein 2a (Slp2a) robustly recruits to the apical membrane where it binds to Rab27a tethered to exocytic Weibel–Palade bodies (WPBs) [84]. PIP_2_ also recruits other apical carriers such as Annexin 2 and PTEN that can locally modify the AMIS to provide a molecular landing pad to tether and dock incoming vesicular traffic [86, 87].

What vesicular cargo is destined to be delivered to the apical membrane during vascular lumen formation(?), and what are the carriers? Definitive studies focusing on post-Golgi carrier’s involvement in AMIS formation and downstream lumen biogenesis are almost completely absent in ECs; however, there is abundant literature detailing proteins that generally affect lumen formation. Podocalyxin is a well-characterized glycoprotein that is one of the first proteins to be transcytosed from the basal surface to the AMIS where it complexes with NHERF1/Ezrin [88]. Podocalyxin is required to initiate cell–cell deadhesion during lumen biogenesis and maintain proper barrier function in ECs [89–92]. As such, podocalyxin is not only regarded as one of the first proteins to be trafficked to the apical membrane, but also a proxy for other required glycoproteins that are delivered at the same time. Although, podocalyxin has been shown to be trafficked by Slp2a, Slp4a, Rab27a, Rab35, Rab8a, Rab11a and others in epithelial cells [45, 93], it is still an outstanding question in endothelial biology. Our data, and others, have demonstrated that Slp2a, Slp4a, and Rab27a have been allocated to WPB trafficking in ECs [83, 84, 94]. Additionally, our unpublished data investigating Rab35, demonstrates that Rab35 is an actin regulator, further signifying disparities between epithelial and endothelial trafficking of podocalyxin. In 2D culture ECs, it has been shown that podocalyxin colocalized with the early endosome marker Rab5 and Rab25 [95]; potentially suggesting a non-Rab11a endocytic or recycling route. As this was a peripheral finding by this group, this association has yet to be confirmed with further experimentation. To our knowledge, there is no singular publication that has comprehensively detailed post-Golgi carriers for podocalyxin in endothelial tissue, and by extension, other important apically targeted glycoproteins. Juxtaposing this finding with epithelial cells highlights the disparity in trafficking-related literature as podocalyxin has been comprehensively investigated in epithelial cyst development.

In the absence of directed ECs studies, we can only speculate as to how apical targeting occurs, leveraging the existing epithelial literature. Many apically targeted proteins such as receptors and sialomucins are heavily glycosylated. This commonality of apical cargo with an inherent heterogeneity of protein domain structures and trafficking-related binding motifs has moved the field away from the idea that every apically targeted protein contains a unique motif that is then recognized by a singular Rab or related effector that would be solely responsible for the delivery of the cargo. Rather, what has come to light more recently is that addition of carbohydrate groups in the acidic TGN can promote oligomerization of apical cargo allowing for a more non-targeted, bulk recognition of this class of proteins by apical carriers [96]. The center piece of this argument is that neutralization of the TGN pH greatly diminishes the delivery of apical cargo, notably glycoproteins such as podocalyxin and p75 [97, 98]. Consequently, the question of what specific Rab may transport podocalyxin is less relevant, but then becomes, what Rab may be responsible for transporting a group of glycoproteins to the AMIS that includes podocalyxin? Rab8, Rab6, and Rab10 have all been implicated as TGN carriers [99, 100], most of which have not been investigated for a role in vascular lumen development. Equally intriguing is the hypothesis that the TGN could play a more regulatory part in staging a bolus release of glycoproteins during lumen formation by differentially regulating its acidity. To this end, the GEF GBF1 has been shown to selectively modulate Golgi transport of anterograde trafficking WPB components in ECs; although its effect on the TGN, per se, has not been tested [101]. This type of signaling could be a developmental control lever for apical membrane-related trafficking; further studies on networks that regulate the aperture of flow through the Golgi are needed. Generally, there are many more questions than answers in the vascular lumen development field. Overtime, it will be interesting to know which programs will display unique organotypic signatures or will be shared between various tissue systems as these trafficking networks are mapped.

## Blood vessel stabilization and Notch trafficking

Central to blood vessel stabilization is the Notch signaling pathway [102]. Although each Notch receptor (1–4) is present in the vasculature, Notch1 is the predominant receptor involved in angiogenesis [102]. ECs with elevated Notch activation adopt a stalk cell phenotype, whereas ECs deficient in Notch signaling will assume a tip cell identity. Notch1 itself, is a transmembrane protein composed of an extracellular domain (NECD) and an intracellular domain (NICD). Importantly, the NECD is composed of 36 epidermal growth factor (EGF) repeats and a negative regulatory region (NRR). The NRR contains three Lin-12-Notch (LNR) repeats that interact with a heterodimerization domain (HD) [103, 104]. Obscured within the interaction between LNR and HD at a resting state is a cleavage site (termed S2). When exposed, the S2 cleavage site is cut by a disintegrin and metalloprotease (ADAM) complex [105]. This cutting event on the S2 extracellular domain precedes cleavage by γ-secretase at the S3 cleavage site to release the NICD. Once freed, the NICD translocates to the nucleus, binding the transcription factor RBPJ/CSL to upregulate downstream genes that promote lateral inhibition [106]. This mechanism necessitates the need for a mechanical force generated by the Notch ligand, Delta-like ligand 4 (Dll4). In this case, Dll4 is presented by the tip cell which pulls on the Notch1 receptor exposing the S2 and downstream S3 domains for cleavage and activation.

How this pulling force is generated is hypothesized to be derived from several scenarios. First, natural cell movement from a leader or tip cell could account for tension needed to separate the LNR and HD domains. Second, and the most reported mechanism, is that upon ligand binding is that Delta undergoes CME (Fig. 1). Two investigations focusing on Dll1 and Notch pulling reported that any perturbation to the CME pathway significantly dampened Notch activation. Using optical tweezers, both groups independently demonstrated that blockade of CME machinery such as epsin, AP-2 or dynamin significantly reduced the pulling force on bead-tethered NECD bound to Dll1 [107, 108]. In endothelial cells, our group demonstrated that CME does not seem to affect Dll4 transcytosis, and presumably pulling forces [109]; thus, it is possible that Dll4 endocytosis is intrinsically different than Dll1, or simply divergent in endothelial tissue. In general, there are currently few studies that have directly looked at Dll4 endosomal pulling forces and Notch activation in endothelial tissue.

With regard to Dll1, it has been shown that Dll1 endocytosis does not require ubiquitination, but ubiquitination is necessary for its recycling back to the plasma membrane and efficient interaction with Notch1 [110]. There is some controversy as others have shown that Dll1 requires ubiquitination to be endocytosed when employing epsin [110]. Regardless, Dll1 has been purported to be contained by a Rab11a recycling loop prior to binding with NECD [111]. Very little has been published directly mapping endothelial-specific Dll4 endocytic mechanisms. Adams et al. demonstrated that synaptojanin-2 binding protein can interact with Dll4 via PDZ binding [112]. In this study, it was hypothesized that synaptojanin-2 binding protein protected Dll4 from lysosomal degradation. Plasma membrane recycling of the other Notch ligand Jagged has been shown to be regulated, in part, by the intermediate filament vimentin [113]. In a more recent investigation, it was reported that Numb acts as a Notch antagonist by controlling the intracellular destination and stability of Dll4 through a post-endocytic-sorting process [114]. Furthermore, Numb negatively controlled the Dll4 plasma membrane recycling through AP1. Given Dll4 is plugged into a Rab11a recycling pathway, it is likely there are other uncharacterized trafficking factors that usher the post-Golgi transport of Dll4 from the TGN to the plasma membrane.

Several reviews on Notch trafficking exist that cover the exocytic and endocytic pathways employed in non-vertebrate organisms [111, 115]; however, in endothelial tissue very little has been published on how Notch is sorted to the plasma membrane or degraded following ligand binding. In other systems, it has long been known that the Notch receptor is ubiquitinated prior to its removal from the plasma membrane [116, 117]. A proteomic approach identified a deubiquitinase called USP10 that functions as an NICD1 deubiquitinase, capable of fine-tuning endothelial Notch responses during angiogenic sprouting [118]. Depletion of USP10 reduced NICD1 abundance and stability and diminished Notch-induced target gene expression in ECs in vitro and in vivo. In a separate investigation, it was shown that RHOQ is essential for the NICD nuclear translocation. The authors report that in the absence of RHOQ, Notch1 becomes targeted for degradation in the autophagy-lysosomal pathway [119]. Testing the interplay between Dll1 and Notch in *Drosophila* neurogenesis, it was found that Dll1 expression induces a quick degradation of Notch in late endosomes. Thus, intracellular trafficking of Notch orchestrates the temporal dynamics of Notch activity [120]. Indeed, it would be interesting to speculate that mechanisms like USP10 are conserved across other Notch pathways. Lastly, it was recently demonstrated how lipid components can interact with Notch trafficking. Shimizu et al. reported that PI3K-C2α is required for the CME of the γ-secretase complex, which allows for the cleavage of endocytosed Notch1 to generate NICD1 in ECs [121]. Overall, there are many unexplored opportunities to further characterize how both Dll4 and Notch are endosomally and exosomally sorted in endothelial tissue, thereby controlling blood vessel stability and homeostasis.

## Secretion in angiogenic development

Due to the endothelium’s role as the primary barrier between the blood constituents and the neighboring tissue, ECs secrete wide swathes of molecules both during development and in adult homeostasis. For the purposes of this review, we will focus on recent reports detailing apical secretion events related to angiogenic blood vessel development. A well-known endothelial-specific secretion mechanism is those that employ WPBs. WPBs are cigar-shaped secretory granules that are primarily found within the endothelium. The most predominant protein housed in this structure is pro-thrombotic von Willebrand factor (VWF), a large multimeric protein capable of initiating the clotting cascade [122]. WPBs are formed at the acidic trans-Golgi and produce their unmistakable shape through folding VWF into a cylindrical structure [123] (Fig. 3). Several reviews go into great depth regarding WPB biogenesis, general trafficking patterns and role in hemostasis referenced here [124–126]. The interesting biology pertaining to WPBs is that their generalized function is entirely contingent on intracellular trafficking.

WPBs have been shown to play other non-clotting related roles required for blood vessel formation. In addition to VWF, more than 183 other proteins have been shown to be associated with WPBs ranging from interleukins to cell surface lectins [127]. The tremendous plasticity of cargo constituents is related to WPBs being a lysosome-related organelle; thus WPBs can be functionally grouped with other structures such as multi-vesicular bodies, melanosomes and secretory lysosomes that regularly intermingle with many other trafficking compartments [128]. Transmission electron microscopy of WPBs shows intraluminal vesicles that contain factors such as CD63, suggesting post-Golgi fusion events can also change the cadre of WPB-house proteins [129]. This finding is exciting as this data suggests the WPB secretory payload could be tailored to match a developmental or homeostatic condition [130].

In angiogenesis, a protein called angiopoietin-2 (Ang2) is secreted via WPB exocytosis. Ang2 can work in both an autocrine and paracrine fashion binding to the Tie-2 receptor. Angiopoietin-1 (Ang1) is outcompeted by Ang2, thus Ang2 was purely considered an Ang1 antagonist [131]. However, more recent evidence has demonstrated that Ang2 can play dual roles in both promoting and repressing blood vessel development [84, 132, 133]. Our lab recently discovered that WPB-mediated exocytosis requires a protein called Slp2a [84]. In the absence of Slp2a, WPBs are still capable of trafficking to the apical membrane, but are not able to fuse, blocking release of WPB cargo. Blockade of WPB-mediated release of Ang2 reduced lumen biogenesis as mentioned above. It is possible the proangiogenic factors galectin-1 or galectin-3 [134, 135] which are also housed in WPBs were mis-trafficked in the absence of Slp2a; however, this was not tested. Other investigations have reported similar findings in which Rab27a, MyRIP, syntaxin-3, synaptotagmin-5, synaptotagmin-like protein-4a, VAMP8, Rab15, Rab33, Rab37, and Rab3d also significantly altered WPB secretion dynamics [94, 136–139]. Of note, the vast majority of the WPB-related trafficking regulators have yet to be tied back to perturbations in angiogenesis, as all studies were primarily conducted in endothelial cells on a 2-dimensional culture dish. Our groups more recent work looking at WPB trafficking in 3-dimensional models both highlight the trafficking and downstream angiogenic ramifications when WPB pathways are perturbed [83].

## Future directions and challenges

In a bulk comparison between epithelial and endothelial studies related to characterizing general trafficking signatures, it is easy to see how little we really understand about how endothelial trafficking events are orchestrated and contribute to physiological and pathological blood vessel development. As mentioned above, a potential reason for this is that epithelial cells exhibit a stereotyped rectangular shape and spatially segregated apical and basal domains allowing for relatively easy imaging of processes at either membrane. Additionally, epithelial cells readily set up apicobasal polarity in 2D culture, thus do not require much in the way of physical or chemical cues to elicit a defined polarity axis [14]. In 2D culture, removed from a sprouting structure, endothelial cells on a dish do not show a commitment to an apical or basal membrane identity. Moving forward, testing 3D sprouting models that provide the necessary cellular cues to reproduce angiogenic morphodynamics with ample sub-cellular imaging will be imperative. Likewise, engineering novel transgenic animals to both visualized vesicular sorting in endothelial cells as well as classic loss and gain of function platforms would significantly aid in our efforts towards identifying novel blood vessel trafficking signatures. Overall, the arena of trafficking-based regulation in endothelial tissues is vast with relatively few full-time occupants. This provides a fantastic research opportunity for truly novel discoveries pertaining to blood vessel biology as well as potential disease therapeutics. We hope to spark many more conversations in the realm of endothelial trafficking as it’s clear that endosomal sorting plays a critically important role in all aspects of blood vessel biology.
